# New Host Records for *Haemoproteus* Spp. (Apicomplexa: Haemosporidiasina) in Passeriformes from North-West of Iran

**Published:** 2017-05-27

**Authors:** Leila Nourani, Mansour Aliabadian, Navid Dinparast-Djadid, Omid Mirshamsi

**Affiliations:** 1 Department of Biology, Faculty of Sciences, Ferdowsi University of Mashhad, Mashhad, Iran; 2 Research Department of Zoological Innovations, Institute of Applied Zoology, Faculty of Sciences, Ferdowsi University of Mashhad, Mashhad, Iran; 3 Malaria and Vector Research Group (MVRG), Biotechnology Research Center (BRC), Pasteur Institute of Iran, Tehran, Iran

**Keywords:** Mosquitos, Hippoboscidae, Ceratopogonidae, *Plasmodium*, Blood parasites

## Abstract

**Background:**

The intracellular protozoan parasites of the genus *Haemoproteus* occur in different avian hosts all over the world. Various genus of blood sucking insects’ families such as Hippoboscidae and Ceratopogonidae could transmit *Haemoproteus* in avian hosts. There are very limited number of studies on wild infected birds with blood parasites in Iran, so the aim of this study was to determine the frequency of *Haemoproteus* spp. infection in passerine birds from northwest of Iran.

**Methods:**

Passerines were collected from four different localities in Zanjan Province, northwest Iran during June to August 2014.

**Results:**

Of 86 passerines, we found *Haemoproteus* infection in 19 (22.09%) individuals. In general, 15 bird species were observed for haemosporidians, of which 53% were infected.

**Conclusion:**

Three species of passerines: *Petronia petronia*, *Sitta tephronota* and *Acrocephalus melanopogon* are new host records for *Haemoproteus* infection in the world. Results acquired by this study support widespread distribution of *Haemoproteus* in passerines and illustrated the prevalence of *Haemoproteus* species in wild birds of northwest of Iran. Conclusively, our study specified that more investigations are needed to reach exact prevalence rate in different families of birds in Iran.

## Introduction

The intracellular protozoan parasites of the genus *Haemoproteus* occurs in different avian hosts all over the world (Bennett et al. 1992, Valkiunas 2005). This genus was originally described by Kruse 1890 as *Haemoproteus columbae* based on gametocytes in *Columba livia*. Haemosporidians as imposing parasites that infect bird’s blood cells in which asexual reproduction stages arise within erythrocytes and sexual cycle occurs in dipteran vectors (Hellgren et al. 2008, Ishtiaq et al. 2008). Various genus of blood sucking insects’ families such as Hippoboscidae and Ceratopogonidae could transmit *Haemoproteus* in avian hosts (Atkinson and van Riper 1991, Ishtiaq et al. 2008, Martinsen et al. 2008). Traditionally, the prevalence of blood parasites is calculated as the amount of infected samples by microscopic detection of blood smears (Peirce 1981). This factor could illustrate transmission degree of the parasites with mosquito’s vectors, richness and also host specificity (van Riper et al. 1986, Hamilton and Zuk 1982). Avian hosts stay diseased if they once become infected by haemosporidian parasites for long time (Valkiūnas 2005).

The Passeriformes with more than 5300 extant species including the largest and most frequently known clades of birds occupied all landmasses except Antarctica (Johanson et al. 2008b). According to recent avian checklist, 235 passerine birds have been recorded from Iran (Dickinson and Christidis 2014).

Parasites have major effects on physiology, ecology, health, population dynamics, sexual selection and fertilization of birds which in some cases lead to host elimination (Hamilton and Zuk 1982, Garvin et al. 2003, Marzal et al. 2005, Donovan et al. 2008), due to very limited number of studies on wild infected hosts with blood parasites in Iran, we have attempted to determine the frequency of *Haemoproteus* species in passerines from northwest of Iran. Besides, new host records were reported for *Haemoproteus*.

## Materials and Methods

Passerine birds were collected from four different localities in Zanjan Province, northwest Iran during June to August 2014. Avian hosts were identified morphologically using field guides (Porter and Aspinall 2010). Blood samples were obtained via brachial vein puncture of the birds with insulin needles. Three thin smears for each specimens were made, immediately air dried, fixed in absolute methanol and finally stained with Giemsa stain pH 7.2 for 45min protozoan detection was done under light microscope for 10–15min at low magnification and then at high magnification (X1000) with immersion oil. Parasites were identified according to the procedures suggested by Valkiunas (2005).

## Results

A total of 86 birds’ belong to nine families were studied. All hosts belong to the order Passeriformes comprising nine families: Turdidae, Paridae, Passeridae, Sittidae, Emberizidae, Fringillidae, Acrocephalidae, Laniidae and Muscicapidae. The highest numbers of captured hosts belong to Fringilidae (Table 1).

**Table 1.** Occurrence of Passeriformes *Haemoproteus* species from Zanjan province, northwest of Iran. Number of examined and infected host is presented. Species marked with star are new reports as *Haemoproteus* infected hosts.

**Host families**	**Host species**	**No. of host captured**	
		**Examined**	**Infected**
**Turdidae**	*Turdus merula*	9	1
**Paridae**	*Parus major*	15	0
**Passeridae**	Petronia petronia*	3	1
	*Passer domesticus*	4	0
**Sittidae**	Sitta tephronota*	2	1
**Emberizidae**	*Granativora melanocephala*	14	3
	*Emberiza buchanani*	1	0
**Fringillidae**	*Linaria cannabina*	14	6
	*Carduelis carduelis*	2	1
**Acrocephalidae**	*Acrocephalus dumetorum*	10	3
	Acrocephalus melanopogon*	5	2
**Laniidae**	*Lanius minor*	1	0
**Muscicapidae**	*Saxicola torquatus*	1	0
	*Irania gutturalis*	1	0
	*Ficedula hypoleuca*	4	0
**Total**		86	19

**Fig. 1.**
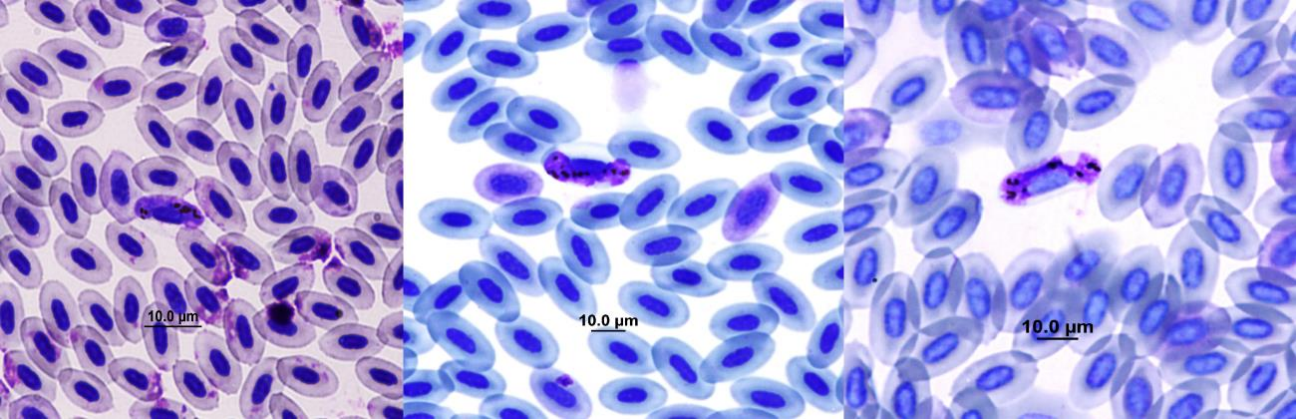
*Haemoproteus* species gametocytes in red blood cells of passerines from northwest of Iran in *Petronia petronia*, *Sitta tephronota* and *Acrocephalus melanopogon* respectively from left. (Photo by L Nourani)

The highest amount of infection was identified in Sittidae (50%) and other families Fringillidae (43.75%), Acrocephalidae (25%), Emberizidae (20%), Passeridae (14.28%), Turdidae (11.11%) were placed in the subsequent orders respectively. Furthermore, families Paridae, Laniidae and Muscicapidae were clear from *Haemoprotues* infection. Besides, new host records were reported for *Haemoproteus* spp. of *Petronia petronia*, *Sitta tephronota* and *Acrocephalus melanopogon* for World (Fig. 1).

## Discussion

This study was designed to discover the occurrence of *Haemoproteus* spp. among different species of Passeriformes. This genus is distributed worldwide with more than 132 species (Valkiūnas 2005). Of 86 passerine birds which examined in this study, we found *Haemoproteus* infection in 19 (22.09%) individuals. In general, 15 bird species were examined for haemosporidians, of which 53% were infected. Results showed three species Passeridae: *Petronia petronia*, Sittidae: *Sitta tephronota* and Acrocephalidae: *Acrocephalus melanopogon* are new host records for *Haemoproteus* infection in the world according to MalAvi database and the rest of hosts’ species infection are also new records for Iran. Till December 2014, about 1129 instances of *Haemoproteus* for 987 different birds’ species was documented around the world.

The most regular genera of intracellular parasites could infect bird hosts including *Haemoproteus*, *Plasmodium* (Marchiafava et Celli 1885) and *Leucocytozoon* (Berestneff 1904). Some investigations reported between 50–100% of the individuals are infected with these blood parasites (Valkiūnas et al. 2003, Belo et al. 2011, Yoshimura et al. 2014) and other researches documented less amount of prevalence (Sehgal et al. 2005, Youssefi et al. 2010, Bahrami et al. 2012, Doosti et al. 2014).

Comparatively, it is little known on blood parasites of wild birds in Iran but, there are numerous studies on Columbiformes (Youssefi et al. 2010, Radfar et al. 2011, Dehghani-Samani et al. 2013, Doosti et al. 2014) and waterfowls (Shemshadi et al. 2014). For example, Youssefi et al. (2010) reported the prevalence of 17.47% for *H. columbae* in north of Iran and examination of 250 pigeons revealed that 20.8% of which were infected with *H. columbae* (Bahrami et al. 2012). In another study from Chaharmahal Va Bakhtiari Province, 220 free living pigeons (*Columba livia domestica*) from five localities presented the prevalence rate of 23.18% for *H. columbae* infection (Doosti et al. 2014). Faghihzadeh Gorji et al. 2012 found prevalence rate of 11% for *Plasmodium* of *Passer domesticus* in Mazandaran Province.

Our report is similar to prevalence rate with the average of 26% for haematozoa in 14812 samples reported for European passerines (Scheuerlein and Ricklefs 2004). High prevalence rate of 39.6% has been recorded for *Plasmodium* infection for 275 passerine birds’ samples of nine families from Brazil by Ribeiro et al. 2004. Likewise another study on 121 birds species captured of West African rainforests, they reported *Haemoproteus* (7.7%), *Plasmodium* (10.7%), *Leucocytozoon* (4.6%) infection (Sehgal et al. 2005).

Migratory birds including Passeriformes and birds of prey are the potential reservoir of various infections which may influence animals and/or man. Birds are able to harbor various pathogens such as chlamydophilosis and salmonellosis agents, poxvirus, *Plasmodium*, feather mite and lice, avian-origin influenza A(H7N9), crimean-congo hemorrhagic fever virus and Lyme disease (Karesh et al. 2007, Vanrompay et al. 2007, Carlson et al. 2011, Lindeborg et al. 2012, Jones et al. 2014). Duo to the importance of transmission of diseases between wildlife and man on public health and economic issues, more studies may shed light on birds and parasites relationship.

## Conclusion

Results support widespread distribution of *Haemoproteus* in Passeriformes and illustrated the prevalence of *Haemoproteus* spp. in wild birds of northwest of Iran. Conclusively, our study specified that more studies are needed to reach exact prevalence rate in different families of birds in Iran.
